# Adverse Events Associated with Administration of Simulation Intravenous Fluids to Patients — United States, 2014

**Published:** 2015-03-06

**Authors:** Misha P. Robyn, Jennifer C. Hunter, Amy Burns, Alexandra P. Newman, Jennifer White, Ernest J. Clement, Emily Lutterloh, Monica Quinn, Chris Edens, Lauren Epstein, Kathy Seiber, Duc Nguyen, Alexander Kallen, Debra Blog

**Affiliations:** 1 Epidemic Intelligence Service, CDC; 2 New York State Department of Health; 3 Division of Healthcare Quality Promotion, National Center for Emerging and Zoonotic Infectious Diseases, CDC

On December 23, 2014, the New York State Department of Health (NYSDOH) was notified of adverse health events in two patients who had been inadvertently administered nonsterile, simulation 0.9% sodium chloride intravenous (IV) fluids at an urgent care facility. Simulation saline is a nonsterile product not meant for human or animal use; it is intended for use by medical trainees practicing IV administration of saline on mannequins or other training devices. Both patients experienced a febrile illness during product administration and were hospitalized; one patient developed sepsis and disseminated intravascular coagulation. Neither patient died. Staff members at the clinic reported having ordered the product through their normal medical supply distributor and not recognizing during administration that it was not intended for human use.

On December 24, NYSDOH and CDC began a collaborative investigation. A review of customer order records identified four additional New York facilities, all outpatient clinical settings, which had received Wallcur simulation saline (Wallcur LLC, San Diego, California) since May 22, 2014, when the company began shipping the product. Staff members at the four clinics reported that they had not intentionally ordered a simulation product and were not aware they had a simulation product until NYSDOH notification; those clinics had not yet administered the product to patients. Two facilities reported receiving an electronic alert that their regular saline product was not available when ordering from their distributor, and were directed to select an alternative. Wallcur manufactures multiple simulation IV products; however, only the simulated 0.9% saline product was reported to have been administered to patients. NYSDOH issued a state health advisory and posted a report on CDC’s Epidemic Information Exchange (Epi-X) to inform public health personnel and medical providers of the potential for inadvertent administration of this product to patients. On December 30, Wallcur issued a request for distributors and customers to return all simulation saline products. Simultaneously, the Food and Drug Administration issued an alert, warning health care professionals not to use any Wallcur simulation IV products in human or animal patients.* On January 6, 2015, Wallcur began a voluntary recall of all its simulation saline products.†

In collaboration with state health departments, CDC conducted a national investigation to assess use of Wallcur simulation 0.9% saline products among patients. Two distributors that had sold the products to clinical facilities were identified. Customer order records from the two distributors revealed that 43 clinical facilities in 23 states had purchased Wallcur simulation saline from the date of first shipment (May 22, 2014) until the date the product recall was initiated (January 6, 2015). All identified clinical facilities were contacted by CDC, or by state or local health departments, informed that Wallcur simulation saline products were not intended for human use, and instructed to observe the product recall. The clinical facilities receiving simulation saline products were outpatient settings, including primary care or family medicine (18 facilities), medical or surgical specialty clinics (17), urgent care (three), rehabilitation or pain clinics (two), chiropractic (two), and clinical research (one). None of the clinical facilities were aware at the time of purchase that this saline product was for simulation and not meant for human use. Ten health care facilities from nine states might have administered the simulation 0.9% saline product to 45 patients (i.e., simulation saline was either administered or reported to be in the facility at the time of saline administration), although the total number of patients nationwide receiving the simulation product is unknown. As of February 9, adverse events had been reported for 25 persons, including 11 hospitalizations. Two deaths occurred among patients administered the product, although it is not known whether the deaths were related to use of the product.

Wallcur simulation products closely resemble IV fluid products intended for clinical use. The bag is labeled “PRACTI-0.9% Sodium Chloride” and the phrase “Practi-Products for Clinical Simulation” is printed in letters <2 mm in height under the Wallcur logo at the bottom of the bag (Figure). No additional warnings or markings on the product indicate that it should not be administered to patients.

This investigation demonstrates the potential for simulation medical products to enter the clinical supply chain, be inadvertently used on patients, and cause harm. This report adds to previously described incidents in which medical training products have been incorrectly used and highlights the potential for future risk to patients (1–3). Health care providers should remain aware that simulation products exist and are reminded to examine the labeling of medical products carefully to ensure that they are intended for human use before purchase and administration. FDA has been working closely with Wallcur to make several changes to its labeling and distribution practices to prevent future occurrences. Further investigation into how simulation products entered the clinical supply chain, including any potential role played by recent national shortages in saline for infusion,§ is ongoing.

## Figures and Tables

**FIGURE:**
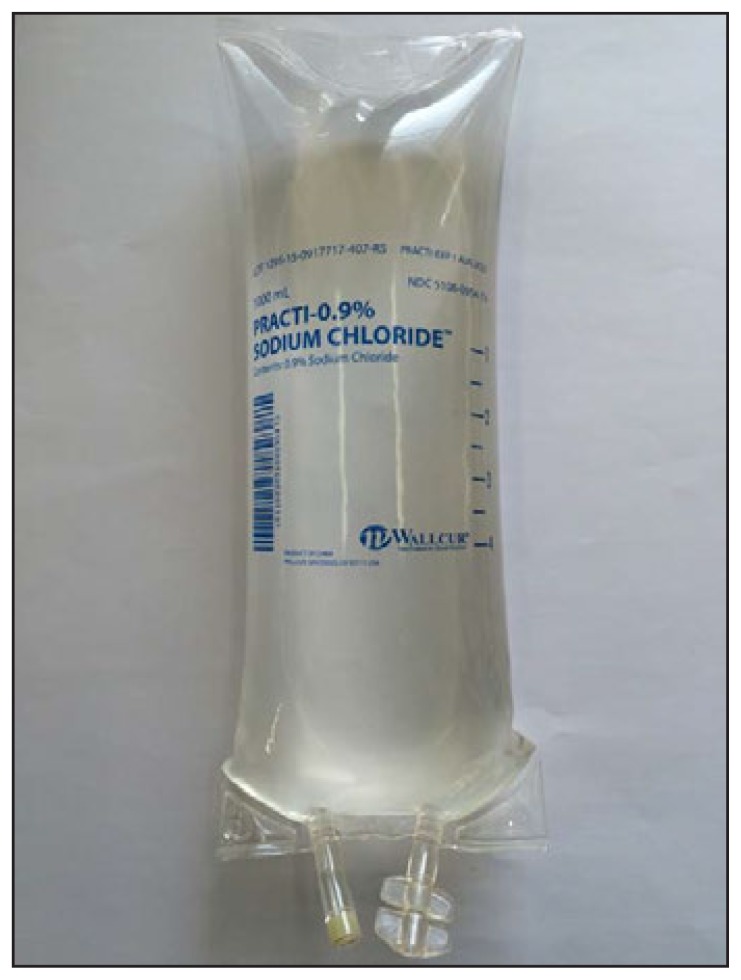
A sample of the simulated saline product inadvertently administered to multiple patients as sterile intravenous fluid, with reported adverse events — United States, 2014

## Footnotes

* Available at http://www.fda.gov/Drugs/DrugSafety/ucm428431.htm.

† Available at http://www.fda.gov/Safety/Recalls/ucm429724.htm.

§ Additional information available at http://www.fda.gov/Drugs/DrugSafety/ucm382255.htm.
